# Phenotyping and hierarchical clustering in genetically unresolved DSD identifies distinct multisystem clinical subgroups—a DSDgene study

**DOI:** 10.1210/jendso/bvag214

**Published:** 2026-09-15

**Authors:** Chrysanthi Kouri, Malika Allimussina, S Faisal Ahmed, Christa E Flück

**Affiliations:** Pediatric Endocrinology, Diabetology and Metabolism, Department of Pediatrics, Inselspital, Bern University Hospital, University of Bern, Bern 3010, Switzerland; Department for BioMedical Research, University of Bern, Bern 3008, Switzerland; Developmental Endocrinology Research Group, University of Glasgow, Royal Hospital for Children, Glasgow G51 4TF, UK; Office for Rare Conditions, Royal Hospital for Children and Queen Elizabeth University Hospital, Glasgow G51 4TF, UK; Developmental Endocrinology Research Group, University of Glasgow, Royal Hospital for Children, Glasgow G51 4TF, UK; Office for Rare Conditions, Royal Hospital for Children and Queen Elizabeth University Hospital, Glasgow G51 4TF, UK; Pediatric Endocrinology, Diabetology and Metabolism, Department of Pediatrics, Inselspital, Bern University Hospital, University of Bern, Bern 3010, Switzerland; Department for BioMedical Research, University of Bern, Bern 3008, Switzerland

**Keywords:** DSD, genetics, organ anomalies, phenotyping, cluster analysis, I-DSD registry

## Abstract

**Context:**

A substantial proportion of individuals with differences of sex development (DSD) remain without an identifiable molecular diagnosis. Systematic phenotyping may reveal clinically relevant patterns and inform genomic investigation.

**Objective:**

To identify phenotypic patterns among individuals with genetically unresolved DSD and determine whether multisystem anomalies form distinct clinical clusters and exhibit overlap with established DSD-associated conditions.

**Design:**

Data from the I-DSD Registry were analyzed for individuals with 46,XY or 46,XX DSD lacking a molecular diagnosis. A 3-part approach was used: (1) unsupervised hierarchical clustering based on Gower distance; (2) supervised phenotype-guided classification using reference profiles for NR5A1/SF-1 deficiency and P450 oxidoreductase deficiency (PORD); and (3) exploratory comparison with Human Phenotype Ontology (HPO)-derived profiles of established DSD-associated genes.

**Results:**

Among 1391 screened individuals, 345 (24.8%) fulfilled inclusion criteria, including the presence of at least 1 additional organ anomaly, 83.8% had a 46,XY karyotype. The most frequent associated findings were small for gestational age (28.1%), cardiac anomalies (21.4%), central nervous system (CNS) (15.7%), and renal anomalies (14.5%). Unsupervised clustering identified 5 phenotypic groups, including craniofacial-dominant and CNS-dominant clusters. Phenotype-guided classification showed overlap with SF-1-deficiency-associated phenotypes in 11% of cases and with PORD-associated phenotypes in 22%. Comparison with HPO-derived profiles of 81 DSD-associated genes demonstrated phenotypic similarities between several clusters and known gene-associated anomaly spectra.

**Conclusion:**

Systematic phenotyping identifies recognizable patterns of multisystem involvement in genetically unresolved DSD. Several clusters overlap with established DSD-associated conditions and gene-specific anomaly profiles, supporting phenotype-driven surveillance, multidisciplinary care, and informing future genomic re-evaluation strategies.

The clinical management of individuals with differences of sex development (DSD) is traditionally guided by clinical presentations and biochemical profiles. However, securing a precise molecular diagnosis remains a cornerstone of precision medicine, as it is essential for understanding heritability, predicting long-term outcomes, and implementing preventive measures (1). While the genetic landscape of DSD has expanded through high-throughput sequencing, a significant “diagnostic gap” persists; in ∼50% of cases, standard genetic analyses fail to yield a definitive molecular cause (2). This persistent diagnostic odyssey underscores the need for novel strategies to unravel the “unresolved” cases and improve clinical management in this patient population.

One such strategy involves the systematic analysis of the clinical phenotype including associated organ anomalies and comorbidities, which are present in ∼25% of individuals with DSD (3, 4). Historically, research has focused on well-characterized conditions such as congenital adrenal hyperplasia, Turner syndrome, and Klinefelter syndrome, uncovering a specific spectrum of associated health issues including cardiometabolic and autoimmune disorders, tumors, and impaired bone health (5-12). In these established cohorts, extragenital anomalies often serve as clue to a specific etiology. For instance, individuals with DSD due to variants in *NR5A1* (SF-1) gene frequently present with lower birth weights and 27% prevalence of associated anomalies, particularly involving the spleen and the central nervous system (CNS) (4). Conversely, DSD due to cytochrome P450 oxidoreductase deficiency (PORD) is characterized by a distinct “phenotypic fingerprint” involving the skeletal system and specific metabolic disturbances (13-15).

The utility of these associated features extends beyond mere clinical description; they represent the pleiotropic effects of genes acting within complex developmental gene regulatory networks (16). When DSD presents as part of a multisystemic syndrome, the anomalies often reflect a disruption in a shared developmental pathway (17). For example, the co-occurrence of DSD with renal malformations may point toward the intermediate mesoderm blueprint originating from the urogenital ridge (18); while skeletal and craniofacial involvement suggests disruptions in the redox machinery or *Hox*-patterning axes that synchronize limb and genital tubercle formation (19-21). Even in “unresolved” cases, these phenotypic patterns may reflect known DSD mimics or point to novel genetic causes that standard pipelines overlook.

Recent advancements in rare disease research emphasize that phenotype-driven approaches, such as deep phenotyping and hierarchical clustering, are critical for improving diagnostic yields (22-24). The integration of standardized phenotypic data, specifically by using Human Phenotype Ontology (HPO)-encoded terms, into genomic and multiomics analyses has been shown to enhance the interpretation of uncertain variants and provide new insights into disease mechanisms (22, 23, 25-28). This is particularly relevant given that patients with genetically “unresolved” DSD may harbor disease-causing variants in more than 1 gene; for instance, 25% of individuals with *NR5A1* variants have likely disease-associated variants in secondary genes, suggesting a frequent oligogenic or synergistic architecture in severe phenotypes (29).

Despite these insights, further research is required to provide clinical guidance for treatment and preventive measures and decode the genetic basis of DSD in the substantial portion of the DSD population that remains unresolved after standard testing. The considerable expansion of the I-DSD registry since the last comprehensive analysis of associated anomalies (3) provides a unique opportunity to investigate a large, international cohort of unresolved cases. By systematically exploring whether distinct patterns of associated organ anomalies cluster within specific DSD types, we can begin to identify the mechanistic basis of these “cold cases.”

By focusing specifically on individuals without a genetic diagnosis, this study aimed to identify recognizable phenotypic clusters that reflect shared developmental pathways or known syndromic DSD mimics. We hypothesized that these clusters could serve as a clinical framework supporting care, highlighting shared developmental pathways, and generating hypotheses for future genomic investigation (1, 30-32).

## Methods

### Ethics and informed consent

The study was approved by the I-DSD Registry Board on the SDMregistries platform (https://sdmregistries.org/). Patient registration in the I-DSD registry adheres to strict ethical guidelines and requires written informed consent. The registry has received overarching ethical approval from the National Research Ethics Service in the United Kingdom as a research database containing data gathered during standard clinical care (19/WS/0131). In Switzerland, national ethics approval was granted with the lead center in Bern (BASEC 2016-01210).

### Patient inclusion

Data from the I-DSD registry were screened on March 24, 2025 to identify eligible individuals based on the following inclusion criteria: (1) a genetically unresolved diagnosis despite standard clinical investigation, (2) a diagnostic category of disorder of gonadal development, androgen action, androgen synthesis/excess, or nonspecific 46,XY DSD, and (3) the presence of at least 1 additional nonsex developmental anomaly. Clinicians of eligible cases were contacted for collaboration approval, and data of their registered individuals extracted for analysis. Collected information included demographics, clinical, and diagnostic details such as center, country, birth data, disorder type, karyotype, genetic findings, and organ anomalies. No further information was requested.

### Data analysis and growth metrics

Descriptive statistics were reported as frequencies/percentages for categorical variables. Standard deviation scores (SDS) for anthropometric measures (birth weight, length, and head circumference at gestational age) were calculated by karyotype using INTERGROWTH-21st reference data (33). One-sample Wilcoxon signed-rank tests assessed median differences between the cohort and the general population, assuming a median difference of zero. Anthropometric SDS were also compared across DSD types using Kruskal–Wallis tests.

Organ anomaly prevalence was compared across the 3 largest DSD types (nonspecific XY DSD, gonadal development disorders, and androgen action disorders). Overall group differences were tested with Pearson's χ^2^; significant results were followed by Fisher's exact pairwise comparisons with Bonferroni correction. A two-tailed *P* < .05 was considered significant.

### Hierarchical clustering and phenotype-driven grouping

A dual-track grouping strategy was applied to investigate the mechanistic basis of unresolved cases. First, to examine the natural clustering of anomalies independent of specific clinical DSD labels, unsupervised hierarchical clustering was performed. We utilized the Gower distance metric to accommodate the binary nature (presence/absence) of the organ anomalies. The resulting dissimilarity matrix was used to construct dendrograms via Ward's minimum variance method. Silhouette analysis was employed to determine the optimal number of clusters, ensuring a balance between mathematical cohesion and clinical interpretability. Analyses were performed on the full cohort and repeated after excluding single-anomaly cases to evaluate if multisystem complexity (≥2 associated organ anomalies) revealed more distinct subgroup patterns informing on possible mechanotypes.

Second, a phenotype-driven classification was performed by aligning anomaly patterns with characteristics of 2 well-defined reference cohorts with genetically proven *NR5A1*/SF-1 variants (4) or *POR* variants (13, 34). The SF-1–like group included individuals with “disorders of gonadal development” or “nonspecific XY DSD” plus anomalies affecting adrenal glands, spleen/blood/lymphatic system, hematological, CNS, and/or small for gestational age (SGA), reflecting SF-1 deficiency (4, 35). The PORD-like group spanned all clinical DSD types and was defined by adrenal, craniofacial, skeletal, respiratory, or eye anomalies, as well as nondefined syndromic presentations, consistent with described PORD characteristics (13-15). A third group comprised all other individuals with DSD that did not fit either phenotype, named “Unclassified.”

### Exploratory genotype–phenotype comparison using HPO annotations

We compiled the major genetic etiologies of isolated and syndromic DSD from 2 large genetic DSD studies (32, 36) and extracted the phenotype-disease annotations of each gene (n = 81) from the HPO database (https://hpo.jax.org/), excluding genitourinary HPO associations. These phenotypes were harmonized and mapped into the organ anomaly categories used in the I-DSD registry (SGA, heart, CNS, renal, craniofacial, skeletal, GI tract, short stature, respiratory, eyes, ENT, nondefined syndrome, skin, blood and lymph, adrenal, hematological, and other) generating a binary gene-by-phenotype matrix indicating which anomalies are known to occur with each gene. We then compared these gene-specific anomaly profiles with the 5 clusters of individuals having ≥2 associated organ anomalies. For each gene, only associated anomalies were retained. For these anomalies, we extracted the corresponding percentage values from each cluster and summed them to obtain a cluster-specific score. The cluster with the highest total score was considered the best phenotypic match for that gene. By integrating all anomalies associated with each gene, this method identified the cluster whose multisystem phenotype pattern most closely aligns with the gene's known clinical spectrum. This analysis was intended to assess phenotypic concordance with established DSD gene-associated anomaly patterns and should not be interpreted as formal biological or molecular validation. Because HPO annotations differ substantially in completeness among genes, similarity scores should be interpreted as exploratory rather than quantitative measures of biological relatedness.

All analyses and visualizations were generated in R (version 4.3.0).

## Results

### Cohort characteristics

Of 1391 genetically unresolved individuals screened in the I-DSD registry, 345 (25%) presented with at least 1 additional organ anomaly and were included in the study (Fig. S1) (37). The median age was 15 years (range 0-67). The majority had a confirmed (n = 289, 83.8%) or presumed (n = 32, 9.3%) 46,XY karyotype; 11 (3.2%) were 46,XX, and 13 (3.8%) were other/unknown. At birth, sex was registered as male in 255 (74%), female in 76 (22%), and unassigned in 13 (4%). Sex reassignment occurred in 20 (5.8%) individuals, primarily among those initially unassigned (Fig. 1A).

**Figure 1:**
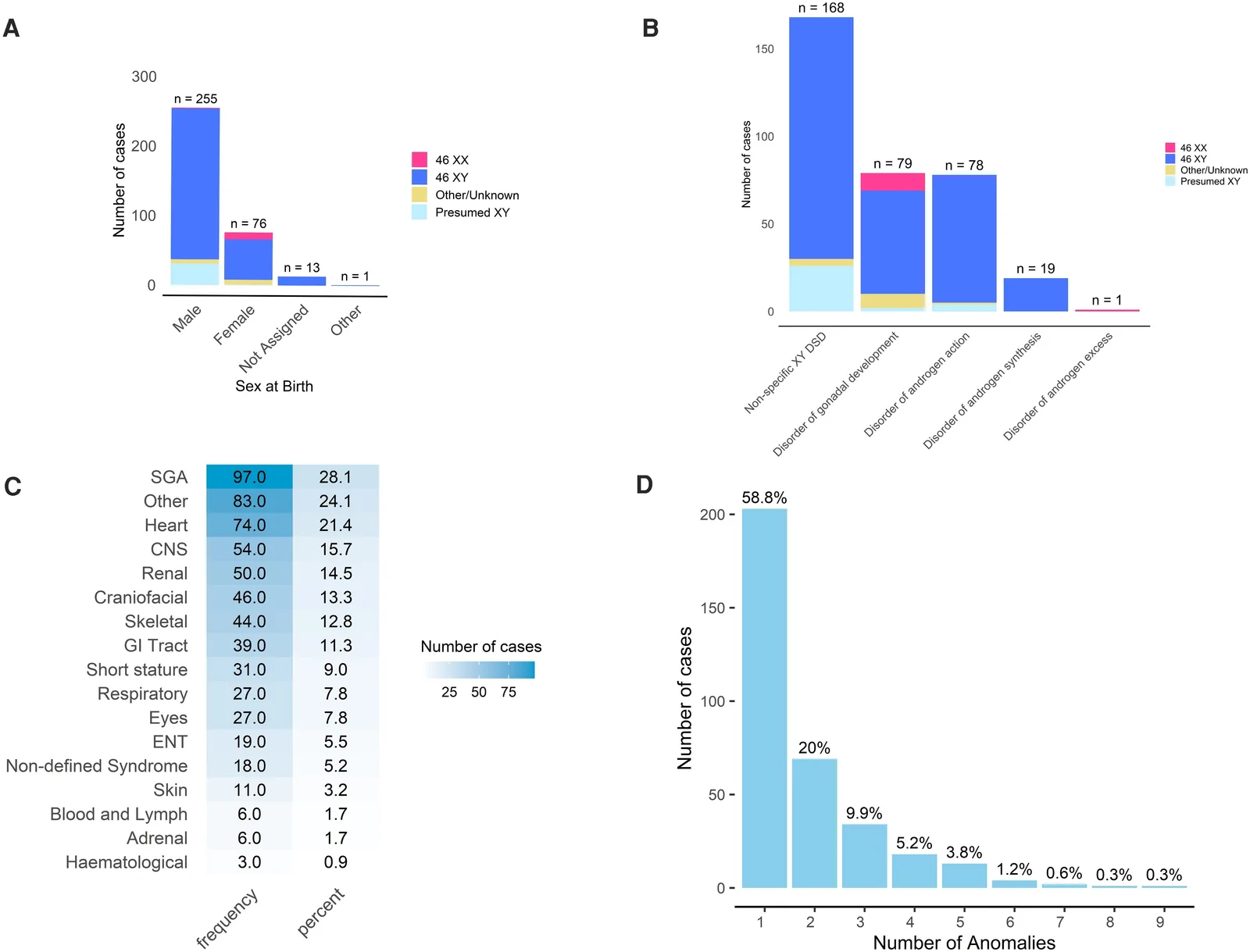
Characteristics of genetically unresolved DSD cases (n = 345). (A) Sex registered at birth stacked by karyotype (B). Distribution of DSD types stacked by karyotype. (C and D) Spectrum of organ anomalies: (C) Heatmap showing the frequency of organ anomalies; individuals with multiple anomalies contributed multiple counts. (D) Bar plot depicting number of cases with one or multiple anomalies.

Clinical DSD classifications (Fig. 1B) included nonspecific XY DSD (168, 48.7%), disorders of gonadal development (79, 22.9%), androgen action disorders (78, 22.6%), and androgen synthesis/excess disorders (20, 5.8%). Median birth weight was 2170 g (SDS −1.75; *P* < .001), length was 45 cm (SDS −1.69; *P* < .001), and head circumference was 31.5 cm (SDS −1.41; *P* < .001). No significant differences in anthropometric measures were observed across clinical DSD types.

### Prevalence of associated organ anomalies

SGA was the most prevalent anomaly (28.1%), followed by unspecified “other” (24.1%), cardiac (21.4%), CNS (15.7%), and renal (14.5%) anomalies (Fig. 1C). Less frequent findings included craniofacial (13.3%), skeletal (12.8%), and gastrointestinal (11.3%) anomalies. Most individuals (58.8%) presented with a single additional anomaly, while 41.2% had multisystem involvement (Fig. 1D); the 46,XY karyotype prevailed (Fig. 1E).

Across the 3 major DSD phenotypes, significant differences were found for SGA (*P* < .001) and GI anomalies (*P* = .005). SGA was more frequent in nonspecific XY DSD (38.1%) and androgen action disorders (30.8%) compared with gonadal development disorders (7.6%). GI anomalies were notably higher in nonspecific XY DSD (17.2%) than in other groups (Fig. 2). Pairwise Fisher's tests confirmed these significant differences (Bonferroni-adjusted *P* < .05) (Table S1) (37).

**Figure 2:**
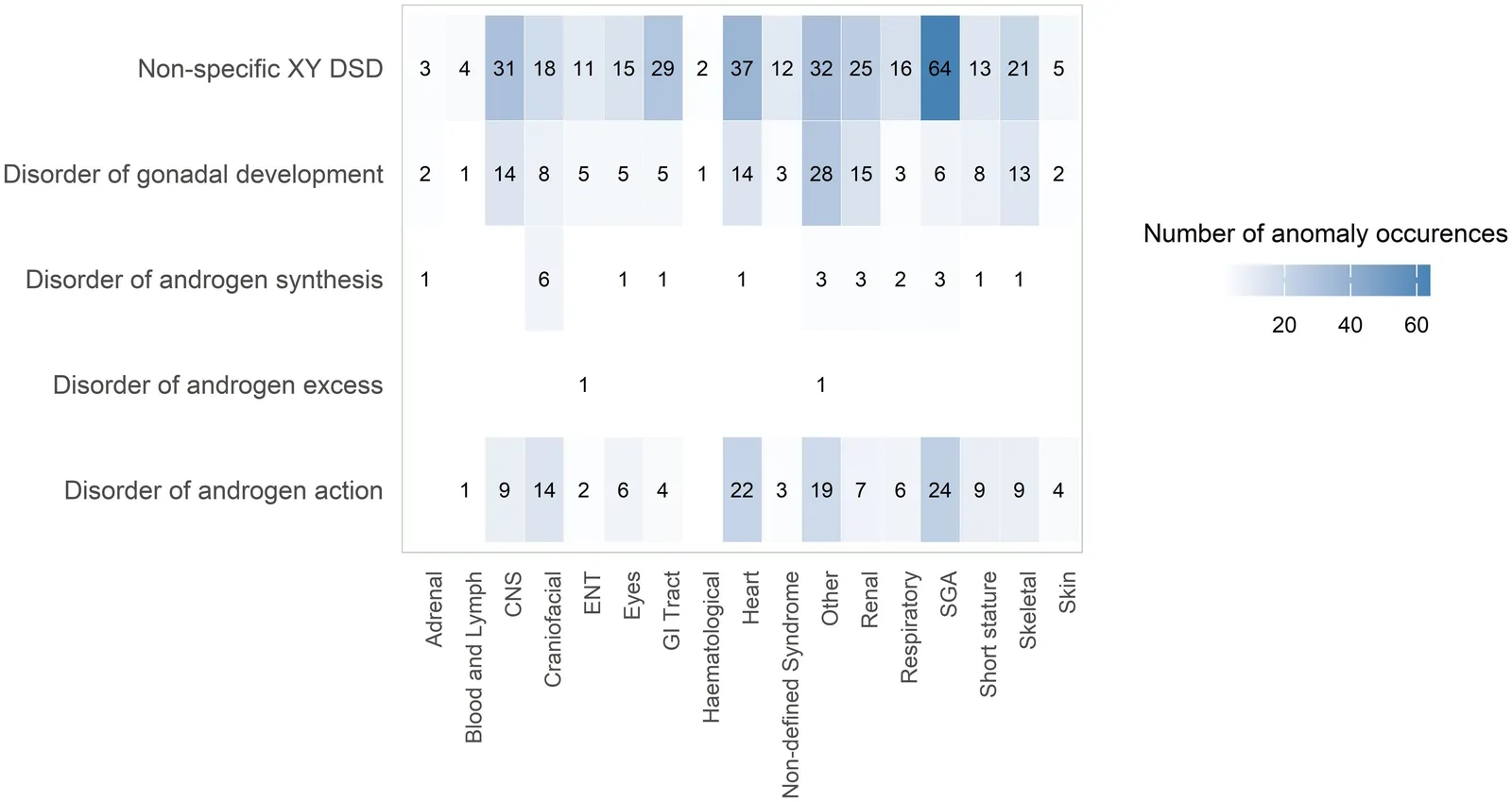
Frequency of different organ anomalies in genetically unresolved DSD cases, grouped by clinically defined DSD types. Heatmap in which each cell indicates the number of a specific organ anomaly reported in individuals with the respective DSD type. Individuals with multiple conditions contributed to multiple cells.

### Clustering of organ anomalies

Unsupervised hierarchical clustering of the full DSD cohort identified 5 phenotypic cluster-groups (Fig. 3A; Table S2) (37). Cluster 1 (n = 48) was defined by “other” anomalies (100%), distributed across all DSD phenotypes. Cluster 2, the largest (n = 178), comprised multiple anomalies (CNS 29%, skeletal 24%, craniofacial 25%, heart 20%, GI 21%, short stature 12%, respiratory 12%, and eyes 14%), and was associated predominantly with nonspecific XY DSD (n = 93) and gonadal development (n = 42), both representing heterogenous DSD phenotypes likely due to genetic variations affecting early fetal development. Cluster 3 (n = 72) was dominated by SGA (99%) and mainly associated with nonspecific XY DSD (n = 47) and androgen action (n = 18). Cluster 4 (n = 24) was renal-dominant (100%), with more gonadal development cases (n = 10). Cluster 5 (n = 23) was cardiac-dominant (100%), spanning all DSD types.

**Figure 3:**
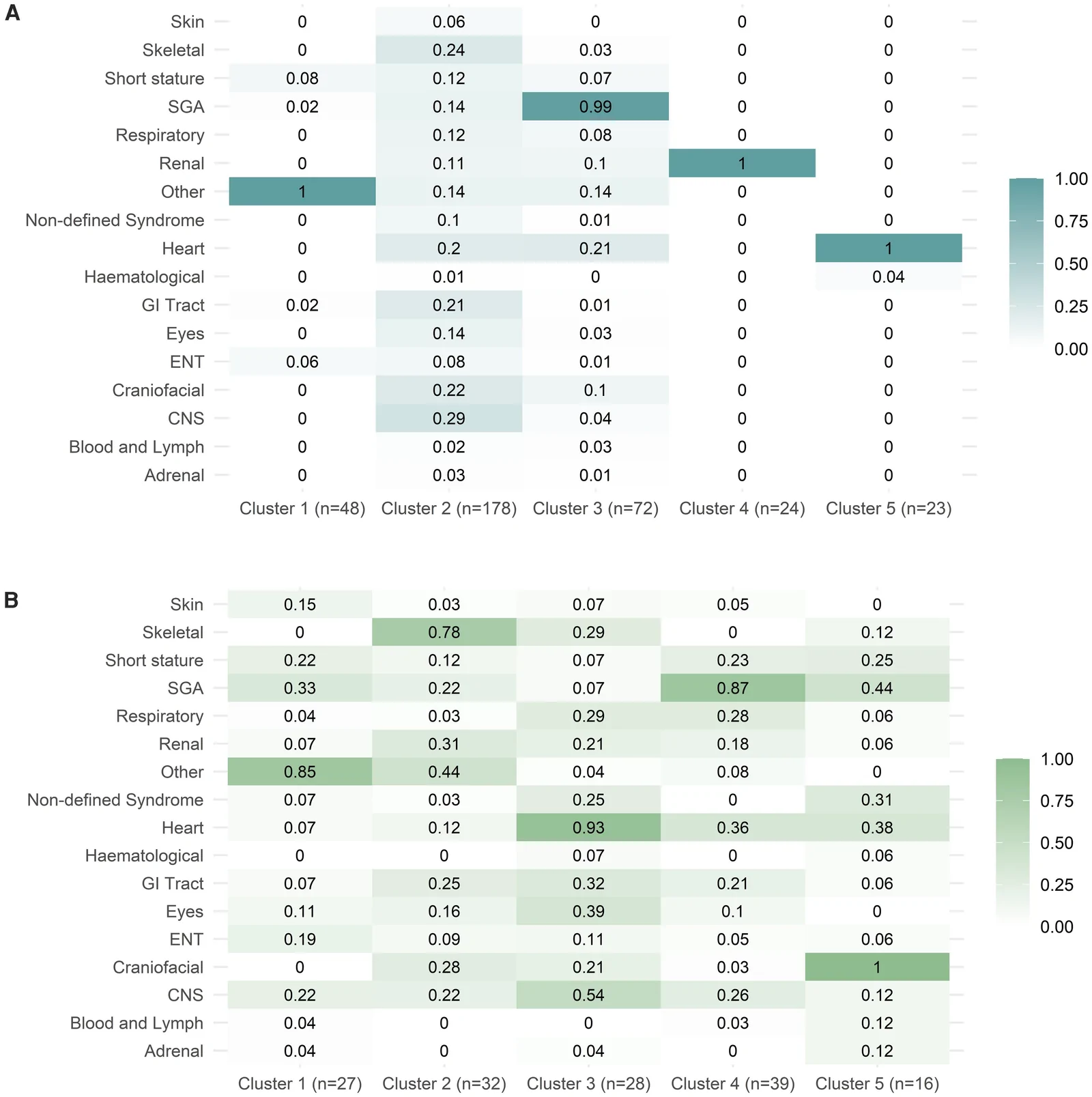
Hierarchical clustering of genetically unresolved DSD cases according to associated organ anomalies. (A) Heatmap including all cases (N = 345). (B) Heatmap with multisystem anomaly clustering (after excluding cases with a single organ anomaly) (N = 142).

To identify more distinct subgroups, we also performed multisystem anomaly clustering on 142 individuals with DSD and ≥2 associated anomalies as the likelihood of co-occurrence by chance might be lower (Fig. 3B; Table S3) (37). Cluster 1 (n = 27) was enriched for “other” anomalies (85%) and SGA (33%), spanning all DSD phenotypes. Cluster 2 (n = 32) was associated with skeletal (78%) and renal (31%) anomalies, more prominent in multisystem cases. Cluster 3 (n = 28) was strongly cardiac (93%), with CNS (54%) and eye (39%) involvement, showing greater specificity. Cluster 4 (n = 39) was also SGA-dominant (87%) but coupled with heart, respiratory, CNS, GI, and renal anomalies, found mainly with nonspecific XY DSD (n = 25) and androgen-related disorders (n = 12). Cluster 5 (n = 16) was craniofacial-dominant (100%), a subgroup not distinct in the full-cohort analysis.

### Phenotype-driven classification

The phenotype-driven classification strategy successfully aligned a third of the cohort with characteristics of 2 established genetic DSDs, SF-1 deficiency (4, 35, 38) and PORD (13-15). Three groups emerged: SF-1-like (N = 34, 10%), PORD-like (N = 79, 23%), and unclassified (N = 234, 67%) (Fig. 4). Two DSD patients met criteria for both classified DSDs and were therefore included in both.

**Figure 4:**
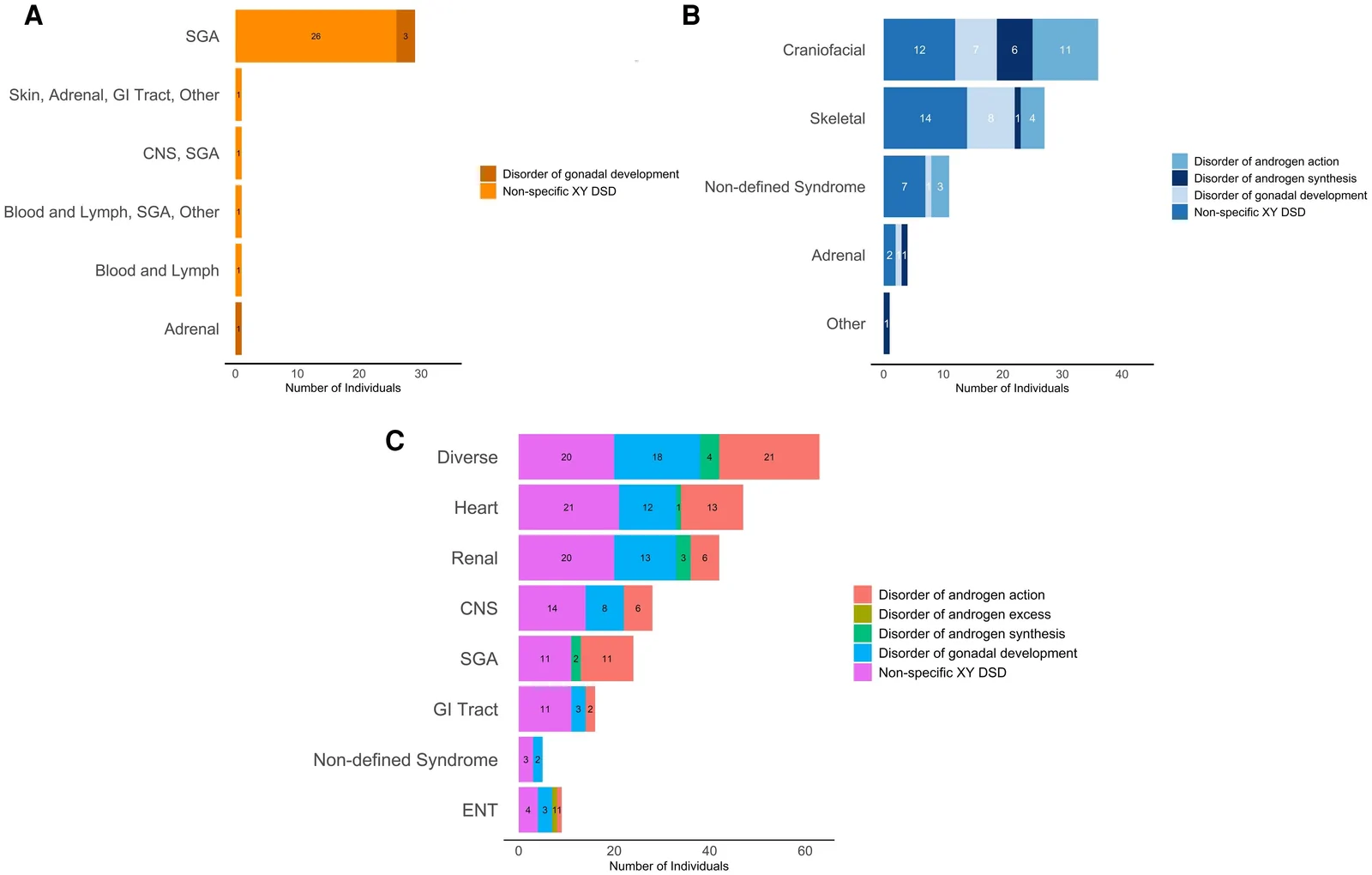
Phenotypic grouping of genetically unresolved individuals with DSD resembling PORD, SF-1 deficiency, or neither. (A) Individuals with a PORD-like phenotype grouped by organ anomalies and stacked by DSD type (N = 79). (B) Individuals with a SF-1-phenotype grouped by organ anomalies and stacked by DSD type (N = 34). (C) Individuals not fitting either phenotype, grouped similarly (N = 234). Although many individuals presented with multiple organ anomalies, each was assigned to a group based on a predefined prioritization of the most prevalent anomalies observed in the cohort to allow for clearer visualization in the plots. The category “Diverse” include less common isolated/not anomalies such as skin, eyes, respiratory, and other.

The SF-1-like group was characterized by “impaired gonadal development” or “nonspecific XY DSD,” adrenal, CNS, spleen/blood/lymphatic anomalies, and SGA features (Fig. 4A). The PORD-like group exhibited impaired steroidogenesis and multisystem anomalies including craniofacial (n = 36), skeletal (n = 27), adrenal, respiratory, and eye anomalies, plus syndromic signs (Fig. 4B), overlapping with clusters 2, 3, and 5 of the multisystem anomalies’ analysis (Fig. 3B). When performing the hierarchical clustering analysis for the unclassified group (N = 234) separately (Fig. 4C), we identified 3 dominant clusters: renal-dominant (n = 24), cardiac-dominant (n = 23), and a novel CNS-dominant cluster (n = 23) (Fig. 5). These clusters became now more apparent as compared with the analysis of individuals with multiple anomalies (Fig. 3B).

**Figure 5:**
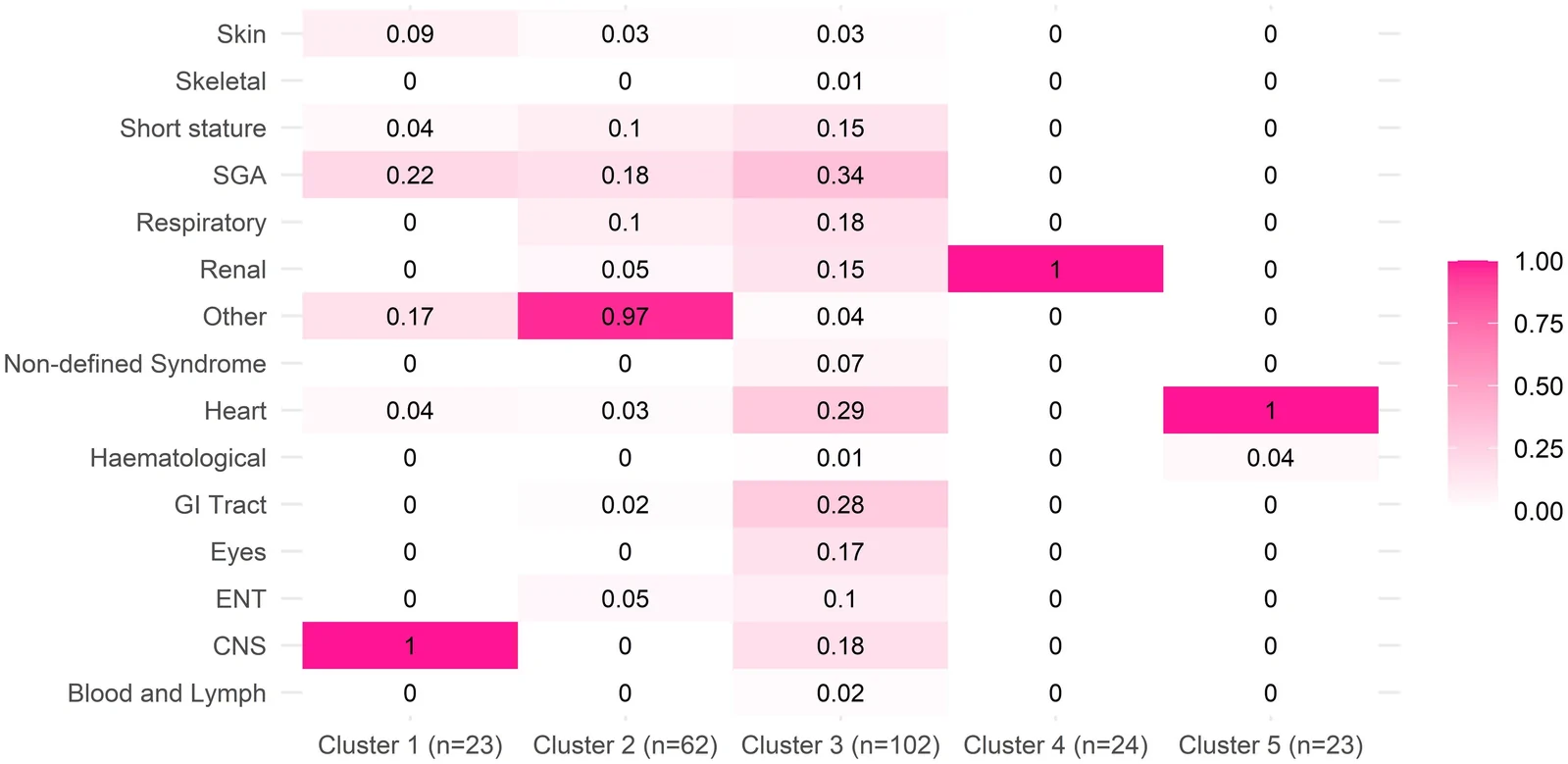
Hierarchical clustering of genetically unresolved DSD cases not resembling PORD or SF-1 deficiency phenotypes. Heatmap including 234 cases of this third group.

### Comparison of multisystem anomaly clusters with phenotype profiles of established DSD genes

Direct molecular validation of the identified clusters was not feasible because individual genomic datasets and biological samples are not available through the I-DSD Registry. We therefore performed an exploratory comparison between the identified phenotypic clusters and phenotype annotations associated with established DSD genes.

Using HPO-derived phenotype annotations from 81 established DSD genes (32, 36), we assessed the degree of phenotypic concordance between published genotype-associated anomaly spectra and cluster-specific anomaly patterns of individuals with ≥2 associated anomalies (Fig. 3B). As shown in the heatmap in Fig. 6, genes with similar anomaly profiles grouped together, forming coherent blocks. Cluster 1, enriched for “other” anomalies and moderate SGA, captured 6 genes (*AMH*, *AMHR2*, *HSD17B3*, *PCSK1*, *RSPO1*, and *SRD5A2*) most of which are associated with moderate to severe DSD, but display a wide range of possible extragenital but less-organ specific anomalies (Table S4) (37). Cluster 2, characterized by skeletal and renal anomalies, associated with 18 genes whose phenotypic characteristics frequently exhibited multisystem but not syndromic presentations (16/18), including several steroidogenic genes. Cluster 3, the cluster with the highest-frequency signals, dominated by cardiac, CNS and ocular anomalies, mapped with the largest proportion of tested genes and phenotypes (54/81), many of them known to be associated with syndromes. Cluster 4, dominated by SGA, did not map to any DSD gene, consistent with the fact that SGA was not a frequent HPO-reported association for established DSD genes, while present in the I-DSD registry data. Cluster 5, defined strongly by craniofacial anomalies, comprised 3 genes (*BMP15*, *CYB5A*, and *FOXL2*) whose associated phenotypes shared craniofacial involvement. These findings suggest phenotypic similarities between several clusters and established DSD-associated gene profiles. Although this analysis does not provide biological validation of the clustering approach, it may offer insights into common underlying gene networks and disease mechanisms.

**Figure 6:**
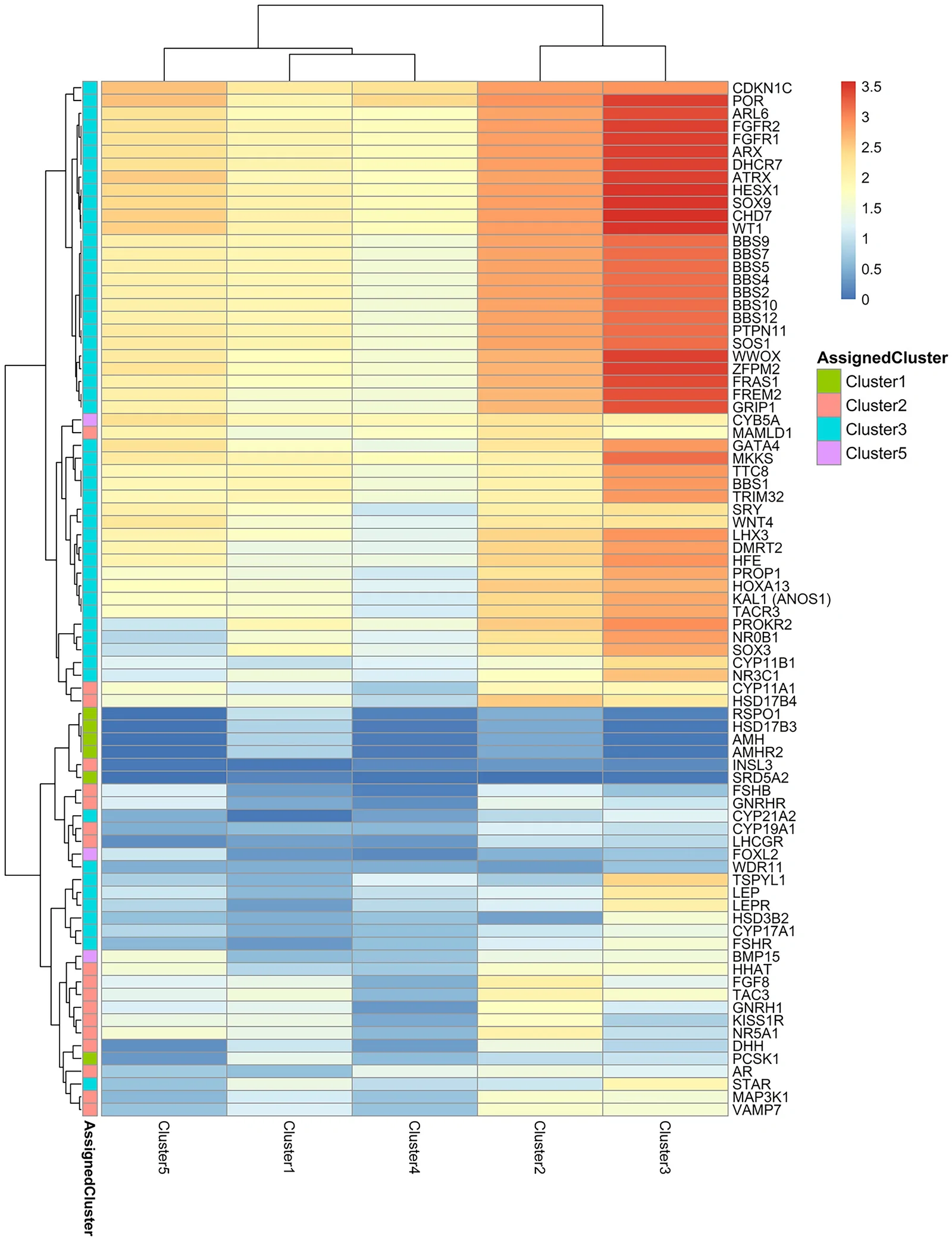
Comparison of phenotype profiles of reported DSD genes with multisystem anomaly clusters identified in genetically unresolved I-DSD cases. The heatmap displays cluster-specific similarity scores calculated for 81 established DSD-associated genes using harmonized Human Phenotype Ontology (HPO) annotations. Each row corresponds to a single gene and each column represents 1 of the 5 anomaly based clusters identified after excluding individuals with only a single associated anomaly (n = 142). Color intensity reflects the degree of phenotypic concordance between the anomaly profile associated with each gene and the observed cluster characteristics. Higher scores indicate greater similarity between the published multisystem phenotype spectrum of a gene and the anomaly distribution within a cluster. This analysis represents an exploratory comparison of phenotype profiles and is not intended as molecular validation of the identified clusters.

## Discussion

In this international cohort of 345 individuals with genetically unresolved DSD and associated organ anomalies drawn from the I-DSD registry, we show that the “unresolved” group is not characterized by a homogeneous collection of isolated endocrine abnormalities, but by a diverse spectrum of multisystemic developmental disorders. Approximately one-quarter of individuals presented with anomalies beyond sex development, a prevalence that mirrors earlier registry-based analyses of the broader DSD population (3). The stability of these frequencies, together with the similar proportions of single vs multiple anomalies, suggests that unresolved cases are not phenotypically distinct from genetically diagnosed DSD. Instead, the persistent diagnostic gap may reflect limitations of current genomic approaches in detecting the full spectrum of pathogenic variation, including noncoding, structural, oligogenic, and regulatory disruptions.

### Phenotypic clustering reveals mechanistically coherent subgroups

By integrating phenotype-guided clustering with established mechanistic frameworks, we identified distinct phenotypic constellations that align with known DSD mimics. Approximately 10-11% exhibited a phenotype resembling the reported anomaly spectrum associated with SF1 deficiency. This constellation is consistent with the pleiotropic role of *NR5A1*/SF-1 in steroidogenic lineage specification, as well as adrenal and spleen development (4, 35, 38). The presence of CNS and spleen/hematological anomalies in this subgroup reinforces the concept that the interactome of SF-1 and SF-1-like factors extends beyond gonadal and adrenal development, reflecting a gene regulatory network with a broader role in early mesodermal and neural patterning. Known genes that could mimic *NR5A1* are *NR0B1* (*DAX1*), *NR2F2*, *PBX1*, *SOX9*, and *LHX9* (39-43).

Similarly, 22-23% displayed phenotypic overlap with the anomaly patterns reported in PORD, dominated by craniofacial and skeletal anomalies. These features are hallmarks of disrupted redox machinery (electron flux) and impaired steroidogenic enzyme activities. The overlap between skeletal morphogenesis and steroidogenesis reflects shared redox and/or cholesterol-dependent pathways and coordinated developmental timing. This subgroup likely harbors variants in “support” proteins or regulatory elements of the steroidogenic network that synchronize appendage formation, craniofacial and skeletal patterning, and genital development (13-15). Genes and pathways that are involved in these processes include HOX clusters, SHH (Sonic Hedgehog), WNT and FGF signaling, retinoic acid pathways, and redox and cholesterol metabolism (19, 20, 44-48).

Yet, two-thirds of the cohort remained unclassified, underscoring the biological heterogeneity of unresolved DSD. For this group, hierarchical clustering revealed renal-dominant, cardiac-dominant, and a newly emerging CNS-dominant cluster (Fig. 5). The latter is particularly notable given parallels with the Deciphering Developmental Disorders (DDD) study, which identified pathogenic variants in chromatin remodelers and transcriptional regulators in individuals with neurodevelopmental disorders, associated with DSD in 8% (49). The overlap suggests that dosage-sensitive regulators of chromatin accessibility and transcriptional timing, which are critical for both neural expansion and early gonadal development, may represent an underexplored area for future genomic investigation in DSD. These findings align with emerging evidence that early embryonic “signaling hubs” coordinate development across multiple organ systems, so disturbances in these shared pathways can lead to specific multisystem anomalies, including atypical sex development. However, unlike for the DDD study, phenotype–genotype validation for the unresolved DSD cohort remains elusive.

### Organ-specific clusters reflect shared developmental origins

Cardiac anomalies were among the most frequent findings in DSD, occurring at rates far exceeding those in the general population (≈0.7%) (50). This enrichment can be explained by the fact that cardiac and gonadal development share dependence on early mesodermal patterning, midline integrity, and common morphogenetic pathways such as SHH, WNT, FGF, and retinoic acid (45, 51). Disruption of these early signaling hubs naturally produces multisystem phenotypes that include, but are not limited to, atypical sex development. Genes such as *GATA4*, *ZFPM2/FOG2*, *NR2F2*, and *MYRF* have established roles in both genetic heart defects and testis determination (52-57).

Renal anomalies are rare in the general population (≈0.3%) (50). However, in line with published literature (58-61), renal anomalies were prominent in our unclassified DSD cohort, particularly in disorders of gonadal development. This is biologically consistent with the shared embryonic origin of the genital and urinary tracts from the urogenital ridge (46). The presence of renal anomalies in unresolved DSD suggests that disruptions in intermediate mesoderm specification or urogenital ridge differentiation may be central to the pathogenesis of these cases. Involved genes include *WT1* (regulatory) and *PBX1* (40, 62). The frequent combination of renal anomalies with cardiac, gastrointestinal, skeletal, and CNS involvement in syndromic DSD reinforces the idea that early developmental regulators with broad, multisystem roles, rather than isolated organ-specific genes, underlie these presentations. These include pleiotropic master regulators (eg, *SOX* family, *WT1*, and *SAMD9*) (62-64), spliceosome/chromatin remodeling (eg, *CHD7*, *ATR-X*, and *ARID1B*) (65-67), and the Hox-patterning axis (eg, HOX cluster and WNT signaling) (48, 68, 69). This is in line with our complementary analysis integrating HPO-based phenotype associations of known DSD genes, which demonstrated that many of them can contribute to multiple anomalies across organ systems and showed concordance with the most-representative clusters. SGA emerged as the most prevalent anomaly, particularly in nonspecific XY DSD and androgen-related conditions. Its association with multisystem involvement is a general observation of all syndromic conditions where an overall prevalence of 10-30% is reported (70-73). For syndromes where DSD is a primary or prominent feature, SGA is a common but not universal feature (74). However, it is highly syndrome-specific and near-obligate in some entities such as MIRAGE syndrome (*SAMD9*) or Silver Russel syndrome (*SRS1*) (75, 76). In MIRAGE syndrome growth restriction reflects global cellular stress, impaired DNA repair, or disrupted transcriptional regulation (75). Thus, in unresolved DSD, SGA may serve as an early clinical marker of severe systemic developmental perturbation causing multiple organ anomalies rather than isolated gonadal dysfunction.

### Comprehensive phenotyping as a framework for clinical characterization and guidance for future investigation

The multisystemic nature of the phenotypic clusters suggests that many unresolved DSD cases may harbor pathogenic variants outside coding regions or within dosage-sensitive regulatory networks not captured by standard exome or panel sequencing. Perturbations in enhancers, chromatin regulators, or transcriptional cofactors, particularly those acting during early embryogenesis, could plausibly disrupt multiple organ systems in parallel (49, 77-79). Our findings therefore support the integration of comprehensive phenotyping with advanced genomic technologies including whole-genome sequencing, long-read sequencing, and multiomics approaches (1, 27, 28, 80).

Although phenotypic clustering does not establish a molecular diagnosis, it provides a structured framework for the clinical characterization of genetically unresolved DSD and helps to prioritize future genomic investigations.

Standardized HPO-based clustering has improved diagnostic yield in other rare diseases (22, 23, 25-28) and our results demonstrate its utility in DSD. For example, when identifying a PORD-like or SF-1-like phenotype, it may be advisable to screen for adrenal insufficiency, bone health issues, or splenic/hematological abnormalities, respectively, even before a molecular diagnosis is confirmed. Conversely, CNS-, renal-, or cardiac-dominant clusters highlight developmental pathways that warrant prioritization during variant interpretation. These phenotype-driven insights may be particularly valuable in cases where genetic findings are ambiguous, such as variants of uncertain significance or oligogenic combinations.

The principal clinical value of the identified clusters lies not in establishing a molecular diagnosis but in supporting anticipatory clinical care. Individuals within cardiac-, renal-, CNS-, or craniofacial/skeletal-dominant groups may benefit from targeted screening for associated organ involvement, multidisciplinary follow-up, and systematic phenotypic documentation. The clustering approach therefore provides a practical framework for risk stratification and clinical management in genetically unresolved DSD. Future studies integrating harmonized genomic data, transcriptomics, and biobanked samples will be required to determine whether these clinically derived clusters correspond to distinct biological entities.

### Limitations

This study is subject to limitations inherent to registry-based data research, including severe selection bias, variability and accuracy in reporting, incomplete longitudinal data, restricted genetic testing information/validation, and potential under-recognition of anomalies emerging later in life.

Importantly, the I-DSD Registry does not currently contain sufficiently detailed or time-resolved information regarding the genetic testing methodologies performed for individual patients. Consequently, it was not possible to determine whether unresolved cases had undergone targeted gene analysis, panel sequencing, whole-exome sequencing, whole-genome sequencing, or subsequent reanalysis. The classification of participants as genetically unresolved should therefore be interpreted in the context of heterogeneous historical diagnostic approaches. Future registry development should incorporate standardized documentation of genetic testing strategies to facilitate genotype–phenotype analyses and reassessment of unresolved cases. Furthermore, clustering results depend on the granularity and accuracy of phenotyping annotations, while clinical heterogeneity persists within clusters. In addition, focusing exclusively on unresolved cases with associated organ anomalies may bias the cohort toward more complex phenotypes, potentially limiting generalizability to the broader DSD population. Finally, in an ideal study, our cluster analyses and data interpretation should be validated through genomic and transcriptomic testing, and/or cross-validated with parallel cluster data of uniformly characterized genetically solved cohorts. However, these tasks require harmonized biomaterial collections and standardized genetic testing of larger cohorts, as well as standardized phenotyping, which were not feasible within this study, but should certainly be attempted in the future. Meanwhile, cluster findings of this study still inform on possible mechanotypes shared with established DSD genes (Fig. 6) and thereby support ongoing genetic analyses, and the prospective collection of these data can help to achieve this goal.

## Conclusion

This study demonstrates that genetically unresolved DSD frequently presents with recognizable patterns of multisystem involvement. Cardiac, renal, CNS, craniofacial/skeletal anomalies and SGA emerged as recurrent phenotypic patterns that form distinct clinical subgroups. Several clusters showed phenotypic overlap with anomaly spectra reported in established DSD-associated conditions, whereas others, particularly the CNS-dominant subgroup, may represent underexplored areas for future investigation. These findings highlight the importance of systematic phenotyping in genetically unresolved DSD and support the use of phenotype-based stratification for clinical surveillance, multidisciplinary care, and prioritization of future genomic studies. Independent molecular validation in cohorts with harmonized genomic data, transcriptomic analyses, and biobanked samples will be essential to establish the biological significance of these clinical subgroups.

## Data Availability

The data that support the findings of this study are maintained within the I-DSD Registry (https://sdmregistries.org/). Due to the rare nature of these conditions and the potential for patient reidentification, the raw data are not publicly available. Deidentified data may be made available to qualified researchers upon reasonable request to the I-DSD Registry Board, subject to ethical approval and a formal data-sharing agreement. All supplementary data supporting this specific study are available at Zenodo: https://doi.org/10.5281/zenodo.21415925.
